# Exploring the Milk Microbiota of Healthy and Mastitic Nili Ravi Buffalo Using 16S rRNA Gene Base Metagenomic Analysis

**DOI:** 10.3390/ani13142298

**Published:** 2023-07-13

**Authors:** Mian Muhammad Salman, Muhammad Nawaz, Tahir Yaqub, Muhammad Hassan Mushtaq

**Affiliations:** 1 Institute of Microbiology, University of Veterinary and Animal Sciences, Lahore 54000, Pakistan; 2 Department of Epidemiology and Public Health, University of Veterinary and Animal Sciences, Lahore 54000, Pakistan

**Keywords:** Nili Ravi, mastitis, milk microbiota, 16S rRNA base metagenomic analysis, diversity indices

## Abstract

**Simple Summary:**

Mastitis is a prevalent issue worldwide among lactating animals. It continues to be a serious problem since it has an economic impact on farmers due to direct and indirect losses. The multifactorial nature of this disease, coupled with the limitations of classical culture-based methods to identify uncultivable bacteria under normal laboratory conditions, exacerbates the situation. In the current study, milk samples from the well-known buffalo breed (Nili Ravi) were tested to explore bacterial communities associated with different udder health statuses using the 16S rRNA gene-based metagenomics technique. The bacterial communities in buffaloes with different udder health statuses, such as healthy, clinical mastitis, and subclinical mastitis, exhibited varied abundance at different taxonomic levels. Additionally, the study concluded that bacterial diversity in milk samples decreases in animals suffering from clinical mastitis compared to those with a healthy or subclinical mastitis status. These findings will contribute to our understanding of bacterial diversity’s role in mastitis development.

**Abstract:**

The Nili Ravi, a buffalo breed from Pakistan, significantly contributes to the dairy industry. Mastitis is one of the major challenges affecting milk production in this breed. The objective of the current study was to identify the bacterial communities and diversity in healthy and mastitic milk of this breed. Milk samples (*n* = 14) were collected from Nili Ravi buffaloes with different udder health statuses, i.e., healthy (5), subclinical mastitis (4), and clinical mastitis (5). The DNAs were extracted, subjected to partial amplification of 16S rDNA (V3 and V4 regions), and sequenced using the Illumina platform. The results revealed variations in the bacterial communities in the milk of animals with different udder health statuses. Proteobacteria was the predominant phylum in the healthy group, while clinical and subclinical mastitis milk had a higher abundance of Firmicutes. Dominant bacterial genera in the healthy group were *Streptococcus* (11.60%), *Herbaspirillum* (7.65%), and *Staphylococcus* (4.70%), whereas the clinical mastitis group was dominated by *Streptococcus* (33.96%), *Staphylococcus* (7.87%), and *Corynebacterium* (2.68%), and the subclinical mastitis group was dominated by *Bacillus* (15.70%), *Corynebacterium* (6.70%), and *Staphylococcus* (6.58%). Assignment of operational taxonomic units at the species level resulted in most species being assigned to uncultured or unknown bacteria or remaining unassigned. Alpha diversity indices indicated lower microbial diversity in the clinical mastitis group, while beta diversity indices showed a scattered pattern of sample clustering in PCA plots among different groups. It is concluded that bacterial diversity in the milk of Nili Ravi buffaloes suffering from clinical mastitis is lower compared to healthy and subclinical mastitis cases. It is concluded that the variations in the microbiota of healthy and mastitic milk may be further investigated and exploited as signature microbes associated with the udder health status of Nili Ravi buffalo.

## 1. Introduction

Buffaloes have been an important choice for farmers among bovines for a long time due to their proficiency in utilizing diets with high roughage content and meeting the demands for animal protein in an escalating global population [1]. Approximately 13% of milk produced worldwide is derived from buffalo [2]. The Asian water buffalo plays a crucial role as an animal resource in 67 countries, providing milk and meat and serving as a source of draught power. It is highly relied upon by people for their livelihood [3]. Buffaloes are an important contributor to the economy of Pakistan, as they account for 68% of the total milk produced in the country. Among the established breeds of buffaloes in Pakistan, the Nili Ravi is a prominent dairy animal, often referred to as “Black gold” due to its high milk production [4]. Milk is an important agricultural commodity for farmers’ incomes and a significant contributor to the livestock sector’s contribution to the national GDP. Pakistan ranks among the top five countries in the world in terms of milk production, with an annual production of approximately 65.74 million metric tons of milk [5]. There is substantial untapped potential for improving the production potential of different dairy breeds in Pakistan, but various challenges impede its progress. One of the significant global challenges faced by the dairy industry, including Pakistan, is mastitis [6,7]. Mastitis caused by antibiotic-resistant bacteria further escalates the problem and increases production losses [8,9,10].

Mastitis is a serious condition that affects one or more quarters of the mammary gland in bovines. It is a multi-etiological inflammatory condition of the mammary gland parenchyma [11,12]. Buffaloes have been considered less susceptible to mastitis than cattle [13], but it still remains a disease that negatively affects the economics of the dairy industry. The cause of mastitis can be multifactorial, with bacteria ranking as a major one [11]. Bacterial agents involved in mastitis can be classified into two major categories: contagious and environmental. Contagious agents include *Staphylococcus aureus*, *Streptococcus agalactiae*, and *Corynebacterium bovis*, while environmental agents include Coliforms (such as *E. coli*, *Enterobacter*, and *Klebsiella*), *Streptococcus uberis*, *Streptococcus bovis*, and *Streptococcus dysgalactiae* [14]. In addition to these mastitogens, the udder microbial community comprises a diverse bacterial population, including both commensal and opportunistic pathogens [15]. Culture-based techniques are considered the primary approaches for identifying the bacteria involved in mastitis. However, negative bacteriological milk cultures can still be used to identify the mastitogens using molecular techniques, i.e., PCR and sequencing [16]. Although molecular techniques, including PCR, sequencing, and metagenomics, are better choices for identifying mastitogens, these still have limited application in field and laboratory setups because of their cost [17]. Although the role of different pathogenic bacteria is known in the development of mastitis, the significance of commensal udder microbiota and its interaction with mastitogens and udder health is less explored and requires further investigation [18].

There is currently limited information available regarding the milk microbiota of buffaloes and its association with udder health. To the best of our knowledge, there is no data on the milk microbiota of Nili Ravi buffalo. Conducting a study in this area would provide valuable insights into the milk microbiota of Nili Ravi buffaloes and the role of different microbial taxa in maintaining udder health. It would also contribute to our broader understanding of various aspects associated with mastitis, including its diagnosis, treatment, and preventive measures. By expanding our knowledge in these areas, we can work towards improving the management and well-being of buffaloes and the dairy industry.

## 2. Materials and Methods

### 2.1. Sample Collection

The sampling for this study was conducted in the Lahore district of Punjab province from May–June 2022. Farms (*n* = 4) were selected, focusing on similarities in husbandry and management practices. Buffaloes between 3 and 6 years of age, having at least one lactation, and not having undergone any antibiotic treatment in the last 15 days were included in the study. Animals with any co-morbidities were excluded from the study. Milk samples for metagenomic analysis were randomly selected from healthy, clinical mastitis, and subclinical mastitis groups (as indicated in Supplementary Tables S1 and S2). The collection of milk samples followed the protocols listed in the National Mastitis Council guidelines [19]. Prior to milk sampling, the udder of each animal was cleaned to remove any attached dust particles and fecal material. Clean water was used to wash the udder, which was then dried with a towel. The udder and teats were physically examined for any signs of inflammation, and a few strips of milk were discarded.

The milk sample was visually observed for texture, abnormal color, and consistency. Aseptically, a 15 mL milk sample was taken in duplicate in sterile Falcon tubes and labeled appropriately. The milk samples were evaluated using the California mastitis test at the farm. The samples were transported to the Probiotics Research Laboratory, Institute of Microbiology, University of Veterinary and Animal Sciences, Lahore, at 4 °C for further processing. The somatic cell count of each sample was conducted using direct microscopic count. The criteria for classifying the samples into healthy, clinical mastitis, and subclinical mastitis groups are given in Table 1. The milk samples were stored at −80 °C until further processing.

### 2.2. DNA Extraction

The laboratory work was conducted in a controlled environment using the same biosafety cabinet, reagents, and kits, all handled by the same person to ensure consistency. Initially, the milk samples were taken out of the freezer and allowed to reach ambient temperature by keeping them at room temperature. A pellet-based method developed by Yap et al. [20] was employed to remove fats from the milk samples. This involved centrifuging 15 mL of the milk sample at 4500× *g* for 20 min at 4 °C and then twice at 13,000× *g* for 1 min at 4 °C. After each centrifugation step, the supernatant was aseptically removed, and sterile phosphate-buffered saline (PBS) was used for washing the retained pellet. DNA extraction was carried out on the pellet and a negative control (PBS) using the DNeasy PowerSoil Kit (Qiagen, Hilden, Germany) following the manufacturer’s guidelines. The extracted DNA was evaluated for purity and concentration (Supplementary Table S3) using a Multiskan Sky microplate spectrophotometer (Thermoscientific, Waltham, MA, USA). Samples with optical density (OD) readings falling between 1.6 and 1.9 at 260/280 nm were considered acceptable for further processing. Samples that met the criteria were labeled appropriately and sent to Macrogen (Seoul, South Korea) for 16S rRNA gene-based metagenomic sequencing.

### 2.3. DNA Quality Control and Sequence Library Preparation

Before sequencing, the quantity of DNA was assessed by processing it through vector 3 fluorometry (Waltham, MA, USA) with the use of DNA binding dye (Invitrogen, cat. #P7589). The 16S rRNA gene was amplified by specifically targeting its V3 and V4 regions by employing forward (TCGTCGGCAGCGTCAGATGTGTATAAGAGACAGCCTACGGGNGGCWGCAG) and reverse primers (GTCTCGTGGGCTCGGAGATGTGTATAAGAGACAGGACTACHVGGGTATCTAATCC) [21]. The library preparation for 2 × 300 bp MiSeq sequencing was performed using the Illumina DNA Prep Kit (Illumina, San Diego, CA, USA). The protocol followed for library preparation was in accordance with the guidelines provided in the Illumina 16S Metagenomic Sequencing Library Preparation Part #15044223 Rev. B document.

Out of the 15 selected samples (5 from each group), 14 successfully passed the library quality control (QC) assessment. However, one sample from the subclinical mastitis group did not meet the QC criteria and was rejected.

### 2.4. Bioinformatics Analysis of Sequences

The bioinformatics analysis was conducted using the Quantitative Insight into Microbial Ecology (QIIME 2) software package, specifically version 2.2020.6 [22]. The 16S rRNA gene sequences (demultiplexed, paired-end reads) were processed following the guidelines provided in the QIIME 2 tutorial. For importing the FASTQ reads, the manifest file import method outlined in the tutorial was utilized. This method allows the import of demultiplexed, paired-end reads into QIIME 2. To filter out noisy data, the denoising method q2-dada2, available as one of the options in QIIME 2, was employed. This method uses the DADA2 algorithm for denoising and quality filtering the sequencing data [23]. The read length chosen for further processing (trimming criteria) was set at 300 base pairs (bp), and any reads longer than this length and chimeric sequences were excluded. Frequency feature tables, representing the frequencies of operational taxonomic units (OTUs), were generated using a specific command-line approach. The assignment of taxonomic labels to the OTUs was performed using a machine learning technique called classify-sklearn, which utilizes a naive Bayes classifier. This classifier was trained using the pre-trained Silva database (Silva 138) and aligned with an identity threshold of 97% to create taxonomic tables [24]. The taxonomic composition of the samples was visualized at various taxonomic levels using heat maps and bar plots. The raw sequences have been deposited in the National Center for Biotechnology Information (NCBI) and are available under the accession number PRJNA977256.

### 2.5. Diversity Analysis

The relative abundance of different taxa at various taxonomic levels was determined by converting the frequencies of OTUs into percentages. To analyze differences in taxa at different taxonomic levels, an analysis of variance (ANOVA) was performed. The results were presented visually using graphs created with Microsoft Excel (Microsoft Corp., Redmond, WA, USA) and GraphPad Prism 8.0.1. (GraphPad Software, San Diego, CA, USA).

To explore microbial diversities, the core metrics phylogenetic method was employed through the QIIME q2-diversity plug-in. Rarefactions were conducted for all samples, setting the thresholds at the minimum value. Alpha diversity was assessed using several indices, including richness and evenness-based measures, such as the Shannon diversity index (H) and Simpson’s index (D), as well as richness-based measures, such as observed features and Chao 1 indices. These indices were compared using the Kruskal–Wallis test.

For beta diversity, which focuses on diversity between samples, various indices were used, including Jaccard distance, Bray Curtis, and weighted and unweighted UniFrac metrics. Principal Component Analysis (PCA) graphs were generated to visualize these metrics. The beta metrics were evaluated using permutational multivariate analysis of variance (PERMANOVA).

## 3. Results

The 14 samples from Nili Ravi milk were divided into three groups: healthy (H), subclinical mastitis (SCM), and clinical mastitis (CM). In total, these samples comprised 142,857 reads, with read counts ranging from 2011 to 8726 reads per sample and a median of 6005 reads per sample. The Healthy (H) group consisted of five samples with a total of 30,652 reads. The subclinical mastitis (SCM) group comprised four samples with a total of 27,586 reads. Lastly, the clinical mastitis (CM) group contained five samples with a total of 25,834 reads. A total of 24 phyla, 78 orders, 131 families, 209 genera, and 84 species in milk samples were detected.

### 3.1. Taxonomic Compositin at Different Level in Nili Ravi Milk

**Figure 1:**
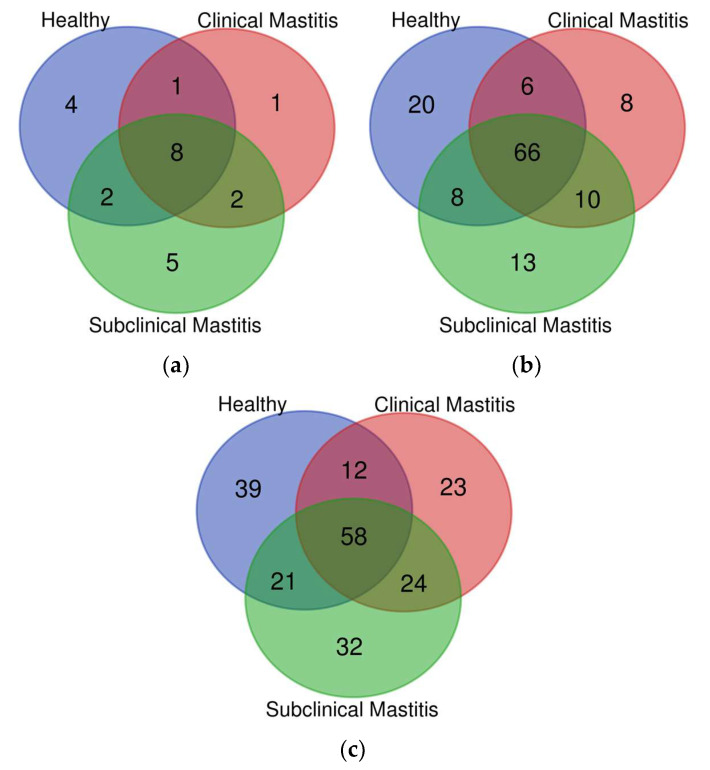
Representation of different taxa (distinct and shared) at different levels: (**a**) phylum, (**b**) family, (**c**) genus, in milk microbiota of Nili Ravi buffalo with different udder health statuses through Venn diagram.

Among the 24 detected phyla, 8 major phyla (Actinobacteria, Bacteroidetes, Cyanobacteria, Firmicutes, Planctomycetes, Proteobacteria, TM7, and Verrucomicrobia) were shared between all groups (H, SCM, and CM). The H group contained four distinct phyla, while the clinical mastitis (CM) group had one distinct phylum (Figure 1a).

**Figure 2:**
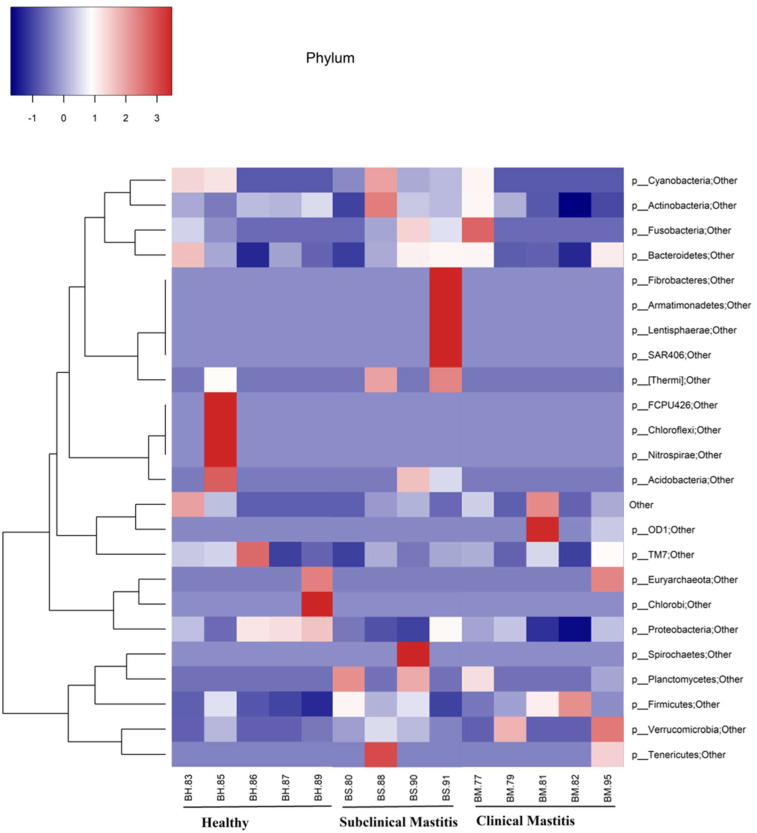
Representation of phylum level taxonomical composition in individual samples through heat map. Intensity of color shows the relative abundance of phyla in milk microbiota of Nili Ravi buffalo with different udder health statuses.

Five major phyla among the 24 detected contributed more than 95% to the overall abundance (Figure 2). The dominant phyla observed in all three groups were Proteobacteria and Firmicutes. In the H group, Proteobacteria was the dominant phylum, while in the SCM and CM groups, Firmicutes dominated the milk microbiota.

**Figure 3:**
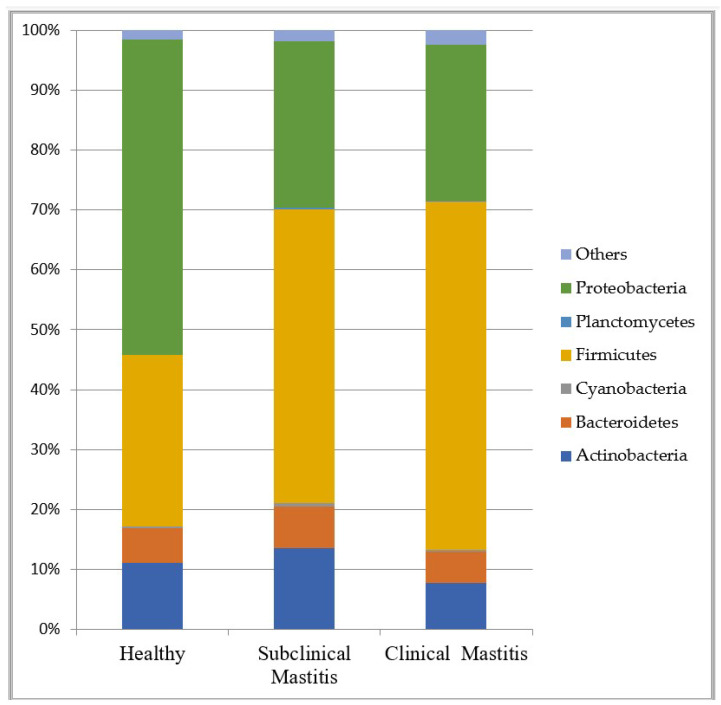
Representation of different phyla across different groups in milk microbiota of Nili Ravi buffalo using taxa bar plot.

The percentage abundance of different phyla across the H group was as follows: Proteobacteria (52.58%), Firmicutes (28.65%), Actinobacteria (11.14%), Bacteroidetes (5.65%), and Cyanobacteria (0.38%). In the CM and SCM groups, a decrease in the abundance of Proteobacteria was observed compared to that of the H group, with abundance values of 26.05% and 27.69%, respectively. The CM group showed a higher abundance of Firmicutes (58.20%), while it was 49.12% in the SCM group. Actinobacteria abundance increased in the case of the SCM group (13.35%), while it decreased in the case of the CM group (7.70%). Bacteroidetes appeared as the fourth-most abundant phyla in all three groups, with the highest abundance recorded in the SCM group, followed by the CM group, and the lowest abundance observed in the H group. The abundance of Planctomycetes was consistently low across all groups (Table 2 and Figure 3).

Among the 66 families shared between the healthy (H), clinical mastitis (CM), and subclinical mastitis (SCM) groups, the H group had 20 distinct families, the CM group had 8 distinct families, and the SCM group had 13 distinct families. There were six families shared between all three groups, eight families shared between the H and SCM groups, and 10 families shared between the CM and SCM groups (Figure 1b). At the family level, the major families detected in all groups were Bacillaceae, Corynebacteriaceae, Lactobacillaceae, Moraxellaceae, Oxalobacteraceae, Staphylococcaceae, and Streptococcaceae (Supplementary Figure S1). The abundance of Bacillaceae (H = 4.22%, CM = 0.87%, SCM = 15.74%) and Lactobacillaceae (H = 1.08%, CM = 0.84%, SCM = 1.88%) increased in the SCM group but decreased in the CM group compared to the H group. Moraxellaceae showed a decreasing trend in both mastitic groups, with a percent abundance of H = 4.22%, CM = 0.98%, and SCM = 3.86%. Corynebacteriaceae had a higher abundance in mastitic groups, with the highest abundance in SCM (6.20%) compared to the H group (1.91%). Oxalobacteraceae showed lower abundance in the mastitic groups (CM = 2.50%, SCM = 2.49%) compared to the H group (9.90%). Staphylococcaceae was more abundant in the mastitis groups compared to the H group (4.52%), with slightly higher abundance in CM (8.21%) compared to SCM (7.52%). The most abundant family detected in the CM group (33.19%) was Streptococcaceae, with a higher abundance compared to the H (11.11%) and SCM (3.95%) groups (Supplementary Figure S1). Moraxellaceae showed a significant decrease (*p* < 0.05) in the subclinical mastitis group, while Oxalobacteraceae showed a significant increase (*p* < 0.05) in the healthy group compared to the other groups (Table 2).

The assessment of microbial diversity at the genus level in Nili Ravi buffalo milk revealed that 58 genera were shared between all groups. The highest number of genera (24) were shared between the mastitic groups, followed by 21 genera shared between the healthy (H) and subclinical mastitis (SCM) groups and 12 genera shared between the H and clinical mastitis (CM) groups (Figure 1c). Distinct genera were found in decreasing order in the H group (39), followed by the CM group (32), and then the SCM group (23). Genera that were detected in high abundance included *Acinetobacter, Bacillus, Corynebacterium, Herbaspirillum, Lactobacillus, Pseudomonas, Staphylococcus*, and *Streptococcus*, accounting for 33.58% of the H group, 48.61% of the CM group, and 40.76% of the SCM group out of the total detected genera (Supplementary Figure S2). At the genus level, *Acinetobacter* was recorded in the highest abundance in the H group (3.40%), while its abundance decreased in the CM group (0.79%) and the SCM group (2.61%). The SCM group was dominated by *Bacillus* (15.77%), *Corynebacterium* (6.70%), and *Lactobacillus* (2.13%), while the CM group was dominated by *Pseudomonas* (1.40%), *Staphylococcus* (7.87%), and *Streptococcus* (33.96%) genera (Table 2).

A high proportion of the species detected in our study fell into the unculturable group. In some cases, no taxonomy could be assigned, and those species were considered unknown. In other cases, the assigned taxonomy was at a higher level, such as uncultured bacterium or bacterium species. The resolution of species classification was not as accurate as other taxonomic levels in our study. Many species were not classified according to specific taxonomic ranks (Supplementary Figure S3). In the H group, important detected species included *Acinetobacter baumannii, Acinetobacter lwoffii, Prevotella nanceiensis, Staphylococcus gelatinosus, Staphylococcus vesicularis, Staphylococcus caen, Streptococcus agalactiae, Bifidobacterium bifidum*, and *Lactobacillus delbrueckii*. In the CM group, low abundances of species such as *Acinetobacter lwoffii*, *E. adolescentis, Prevotella nanceiensis, Sciuri species, Staphylococcus xylosus, Staphylococcus australicum, Streptococcus agalactiae, Streptococcus dispar, Streptococcus dysgalactiae,* and *Lactobacillus helveticus* were detected. In the SCM group, species like *Acinetobacter granulosus, Acinetobacter lwoffii, Citrobacter hathewayi, Prevotella nanceiensis, Sciuri* sp., *Staphylococcus australicum, Staphylococcus caeni, Streptococcus agalactiae, Streptococcus dysgalactiae*, and *Lactobacillus helveticus* were detected, but in low abundance. It is important to note that the species assignments and their abundance varied between the groups, and there were instances where species could not be classified at specific taxonomic ranks, such as phylum, class, order, and genus.

### 3.2. Differnces in Alpha and Beta Diversity Indices in Healthy and Mastitic Groups

#### 3.2.1. Alpha Diversity Indices

**Figure 4:**
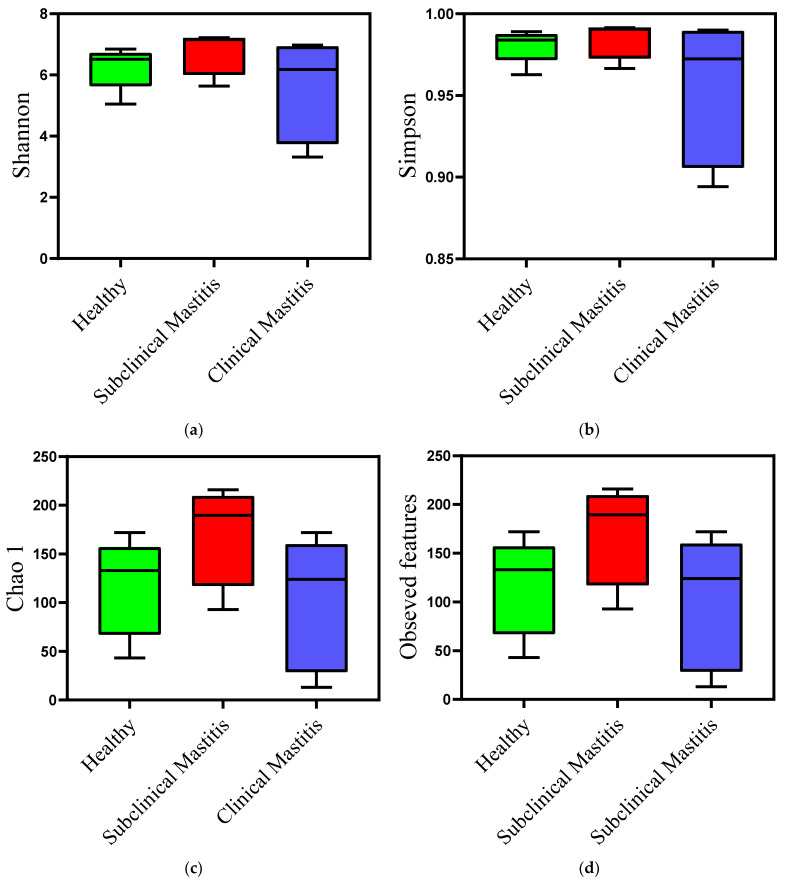
Diversity analysis using alpha diversity indices of microbiota of Nili Ravi buffalo milk with different udder health statuses: (**a**) Shannon diversity index, (**b**) Simpson diversity index, (**c**) Chao 1, (**d**) observed features.

Milk samples from animals diagnosed with clinical mastitis showed a reduction in both types of diversity indices compared to those of the healthy group. This reduction was observed numerically and graphically, indicating a decrease in microbial diversity. However, the difference was not statistically significant (Table 3). In contrast, the milk samples from the SCM group showed a slightly different trend. The Shannon and Simpson diversity indices exhibited a slight difference compared to the healthy group, suggesting a potential dysbiosis of the udder in this type of mastitis (Figure 4). When compared to the CM group, the indices were higher in the SCM group, but the difference was not statistically significant.

#### 3.2.2. Beta Diversity Indices

**Figure 5:**
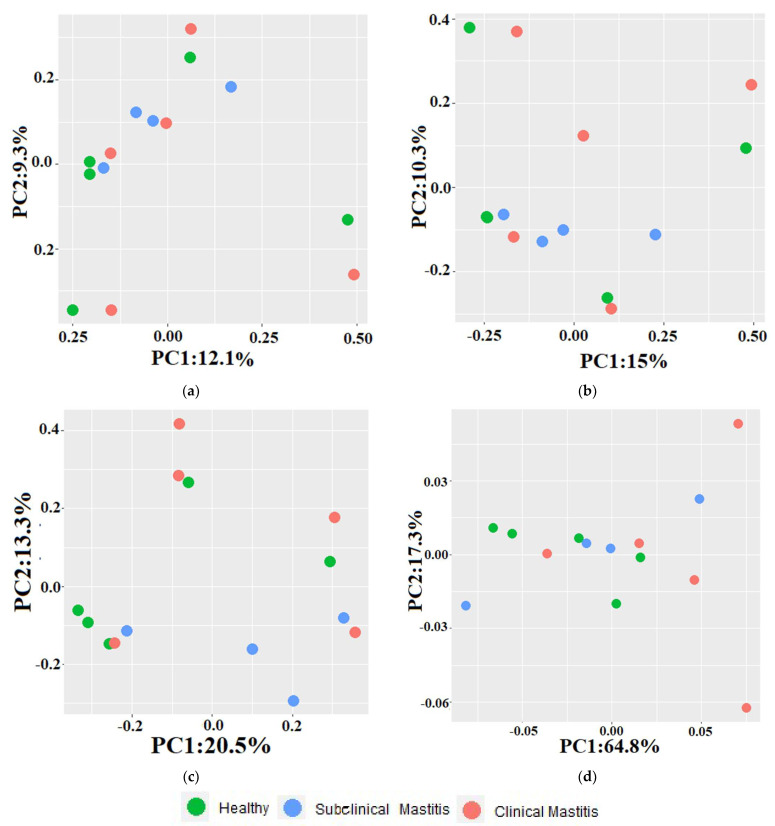
PCA plots representing beta diversity indices in Nili Ravi milk with different udder health statuses: (**a**) Jaccard index, (**b**) Bray-Curtis dissimilarity index, (**c**) unweighted, (**d**) weighted uniFrac metrics.

The differences observed between the healthy and mastitic groups using beta diversity indices are depicted in the principal component analysis (PCA) plots (Figure 5). Across all indices, a scattered pattern was observed in the PCA plots. Principal component 1 (PC-1) and principal component 2 (PC-2) accounted for 9.3% and 12.1% of the variation in the Jaccard index, respectively. In the Jaccard index PCA plot, 40% of the H samples clustered with 75% of the SCM samples and 40% of the CM samples. The remaining samples exhibited a scattered pattern, with H and CM samples clustering along the axis at different points. The PC-1 and PC-2 contributed 10.3% and 15.1% to the total variation in the Bray–Curtis dissimilarity index PCA plot, respectively. In this plot, 60% of the samples were relatively far from the H group samples. For the unweighted UniFrac distance metric, PC-1 accounted for 20% of the variation, and PC-2 accounted for 13.3% of the variation. In the weighted UniFrac distance metric, PC-2 contributed 64.8% of the variation, while PC-1 contributed 17.3% of the variation. In the unweighted metric PCA plot, H samples (60%) clustered with SCM samples (25%) and M samples. In the weighted metric PCA plot, a mixed pattern of sample clustering was observed, with 60% of the samples from the CM group being comparatively far from the H group samples.

## 4. Discussion

The characterization of milk microbiota in cattle and buffalo breeds in Pakistan using the 16S rRNA gene-based metagenomics technique still needs to be explored. To the best of our knowledge, this study represents the first attempt to investigate the milk microbiota specifically in the Nili Ravi Buffalo breed. The multifactorial nature of mastitis, including the interplay between microorganisms and host defense mechanisms, as well as the potential influence of environmental factors on microbial dysbiosis and disease development, necessitates an exploration of the unculturable microbiota associated with mastitis. Traditional techniques relying on culture-based identification methods have limitations, as they can only identify approximately 1% of microorganisms, while the remaining 99% of bacteria are considered unculturable [25]. The development of next-generation sequencing techniques has opened new horizons for insight into the microbiota of different habitats by allowing the sequencing of DNA or RNA of microbes from complex samples [26,27].

In this study, we investigated the milk microbiota in relation to udder health status, including healthy, clinical mastitis, and subclinical mastitis, utilizing the 16S rRNA gene-based metagenomics technique. The selection of samples was conducted from farms having similar management and husbandry practices to minimize the potential impact of these variables on the results. Firmicutes was the dominant phylum in the clinical and subclinical mastitis groups, which is consistent with previous findings [28,29]. This study reports a higher abundance of Proteobacteria in the healthy group compared to clinical and subclinical mastitis groups, which is in contrast to a previous study [30]. A higher abundance of Proteobacteria can be attributed to common environmental pathogens, i.e., *Escherichia, Klebsiella, Pseudomonas,* and *Enterobacter* [31]. Although these Proteobacteria are considered mastitogens, their frequent isolation from healthy milk samples indicates their opportunistic nature and highlights the complexity of their involvement in mastitis [32].

At the family level, Staphylococcaceae and Streptococcaceae were detected in all three groups, but a trend of higher abundance of both of these families was observed in the clinical mastitis group since the members of these families are major etiological agents of mastitis [33,34], and their higher abundance has been previously reported [35]. Moraxellaceae have been observed in higher percentages in both healthy and subclinical mastitis samples. Previous studies have reported the presence of Moraxellaceae in milk and feces on farms, suggesting the possibility of transmission from feces to milk [36]. The observed pattern at the family level is consistent with the genus level analysis, particularly for *Streptococcus*, which was found to be more prevalent in the clinical mastitis and healthy groups compared to the subclinical mastitis group. Since *Streptococcus* is a diverse genus containing many commensal and pathogenic organisms, their abundance in healthy and clinical mastitis groups is not surprising. Discrimination at the species level can provide a better understanding of the complex role of commensal streptococci in the progression of mastitis [15]. Furthermore, the high abundance of streptococci in the healthy group indicates that some of the streptococci species may live as commensals in the udder.

Various species of *Streptococcus* and *Staphylococcus* were detected in all groups. However, due to limitations in resolution and classification, many species were assigned to uncultured and unknown taxa, which hampers a comprehensive understanding of the species composition. In line with a previous study, on bubaline mastitis, the presence of the *Acinetobacter* genus was observed in both healthy and subclinical mastitis samples [37]. The *Acinetobacter* genus is known to include opportunistic species that have been observed at the teat apex [38]. Although different species of *Acinetobacter* have previously been reported from mastitic samples, these are not considered common etiological agents [28,39]. In the current study, *Pseudomonas* was only detected in a few samples from the subclinical group. *Pseudomonas* has been reported as an etiological agent of mastitis in various domestic dairy animals [40].

The assessment of diversity within the groups using alpha diversity indices revealed that the clinical mastitis group exhibited lower diversity compared to the other groups, which aligns with findings from previous studies conducted on bovines [37,41]. Interestingly, in our study, the subclinical mastitis group exhibited the highest diversity, which is in contrast with previous findings in cattle and buffalo, where subclinical mastitis milk showed lower alpha diversity indices compared to those of healthy animals [30,37]. However, a study conducted on goats, where alpha diversity was assessed based on different udder health statuses, reported a similar pattern to what is observed in the current study and suggested that the higher diversity in subclinical mastitis samples is attributed to a slight dysbiosis leading to an imbalance in the microbial community [42]. The beta diversity indices in our study exhibited a mixed pattern, with no clear separation observed between the samples from different groups. These findings are consistent with those of previous studies conducted on goats and cattle [42,43,44]. The lack of distinct clustering suggests that there is an overlap in the microbial composition among the different groups, indicating potential similarities or shared microbial dynamics across udder health statuses.

## 5. Conclusions

It is concluded that the milk of healthy and mastitic Nili Ravi buffalo contains complex and diverse microbial communities. There is a reduced microbial richness in mastitic milk, which may indicate a possible role of other normal inhabitant and commensal bacteria in mastitis. The variations in microbiota of healthy and mastitic milk may be further investigated and exploited as signature microbes associated with the udder health status of Nili Ravi buffalo.

## Figures and Tables

**Table 1:** Allocation criteria for samples into different groups.

Udder Health Status	Clinical Signs	Milk Characteristics	CMT	SCC
Healthy	Absent	Normal	Negative	Below 200,000 cells/mL
Subclinical Mastitis	Absent	Normal	Positive	Above 200,000 cells/mL
Clinical Mastitis	Present	Abnormal	Positive	Above 200,000 cells/mL

Clinical Signs: inflammatory signs in udder, milk characteristics = abnormalities in milk, CMT = California mastitis test, SCC = somatic cell count.

**Table 2:** Comparison of mean (% abundance) at different levels in healthy (H), subclinical mastitis (SCM) and clinical mastitis (CM) groups of Nili Ravi buffalo milk. Only significantly different taxa are denoted with letters.

Taxa	Healthy(H)			Subclinical Mastitis (SCM)			Clinical Mastitis (CM)		
Phylum	Mean	SEM		Mean	SEM		Mean	SEM	
Actinobacteria	11.14	0.94	ns	13.55	4.49	ns	7.70	2.88	ns
Bacteroidetes	5.65	2.37	ns	6.91	2.25	ns	5.32	2.25	ns
Cyanobacteria	0.38	0.23	ns	0.56	0.23	ns	0.16	0.16	ns
Firmicutes	28.65	8.28	ns	49.12	11.12	ns	58.20	13.02	ns
Planctomycetes	0.00	0.00	ns	0.31	0.18	ns	0.11	0.09	ns
Proteobacteria	52.58	8.74	ns	27.69	10.06	ns	26.05	8.88	ns
Family	Mean	SEM		Mean	SEM		Mean	SEM	
Bacillaceae	4.22	1.47	ns	15.74	15.23	ns	0.87	0.31	ns
Corynebacteriaceae	1.91	0.39	ns	6.20	3.89	ns	2.42	1.38	ns
Lactobacillaceae	1.08	0.52	ns	1.88	1.48	ns	0.84	0.61	ns
Moraxellaceae	4.28	0.76	a	3.86	0.80	b	0.98	0.43	a
Oxalobacteraceae	9.90	2.43	a	2.49	0.44	b	2.50	0.93	b
Staphylococcaceae	4.52	1.03	ns	7.52	4.80	ns	8.21	4.30	ns
Streptococcaceae	11.11	7.74	ns	3.95	1.81	ns	33.19	18.82	ns
Genus	Mean	SEM		Mean	SEM		Mean	SEM	
*Acinetobacter*	3.40	0.49	a	2.61	0.78	b	0.79	0.42	ab
*Bacillus*	1.71	0.88	ns	15.77	15.39	ns	0.38	0.38	ns
*Corynebacterium*	2.09	0.40	ns	6.70	4.21	ns	2.68	1.51	ns
*Herbaspirillum*	7.65	3.73	ns	2.06	0.69	ns	0.61	0.38	ns
*Lactobacillus*	1.16	0.60	ns	2.13	1.70	ns	0.91	0.66	ns
*Pseudomonas*	1.27	0.71	ns	0.62	0.50	ns	1.40	0.91	ns
*Staphylococcus*	4.71	1.04	ns	6.58	4.20	ns	7.87	4.60	ns
*Streptococcus*	11.60	8.02	ns	4.29	1.90	ns	33.96	18.78	ns

Different letters (a,b) in the same rows denotes differences between means for *p*-value < 0.05. ns = non-significant.

**Table 3:** Bacterial diversity analysis using alpha diversity indices in healthy, subclinical, and clinical mastitis milk of Nili Ravi buffalo.

Alpha Diversity Index	Healthy (H) mean ± SEM	Subclinical Mastitis (SCM) mean ± SEM	Clinical Mastitis (CM) mean ± SEM	*p*-Value
Shannon diversity index	6.242 ± 0.3150	6.790 ± 0.384	5.506 ± 0.7461	0.1481
Simpson diversity index	0.9805 ± 0.0047	0.9849 ± 0.0061	0.9526 ± 0.01976	0.1481
Chao1 index	116.2 ± 22.42	172 ± 27.06	100.2 ± 30.63	0.1408
Observed features	116.2 ± 22.42	172 ± 27.06	100.2 ± 30.63	0.1408

*p*-values were considered significant at *p* = 0.05, 0.01, 0.001.

## Data Availability

The datasets generated and/or analyzed during the current study are available in the National Center for Biotechnology Information (NCBI) under accessions number PRJNA977256 (https://www.ncbi.nlm.nih.gov/bioproject/?term=PRJNA977256).

## Supplementary Materials

The following supporting information can be downloaded at: https://www.mdpi.com/article/10.3390/ani13142298/s1; Figure S1: Representation of family-level taxonomical composition in individual samples through heat map; Figure S2: Representation of genus-level taxonomical composition in individual samples through heat map; Figure S3: Representation of species-level taxonomical composition in individual samples through heat map; Table S1: Farm data from which samples were collected; Table S2 Animal data from which sample collection was conducted. Table S3: Concentration of extracted DNA from milk samples.
