# Supplementary material for: Exploring the Milk Microbiota of Healthy and Mastitic Nili Ravi Buffalo Using 16S rRNA Gene Base Metagenomic Analysis
Source: Animals (Basel). 2023 Jul 13;13(14):2298. doi: 10.3390/ani13142298 (PMC10376726; doi:10.3390/ani13142298)
Supplement: Supplementary file 1 [file animals-13-02298-s001.zip › animals-2457324-supplementary.pdf]

Supplementary Table S1: Farm Data from which samples were collected

Dairy Farm Reference ID	Farm location	Total Buffaloes	M	F	less than 1 year	1-3 years	above 3 years	Stall Feeding			Milking Practice	Floor type	Cleaning of animal	Milking parlor condition
								green fodder	commercial feed	Wheat Straw				
I	Lahore	20	1	19	4	1	15	yes	Yes	Yes	Hand Milking	Concrete	Daily	clean
J	Lahore	21	1	20	4	2	15	Yes	Yes	Yes	Hand Milking	Concrete	Daily	clean
k	Lahore	19	1	18	3	2	14	Yes	Yes	Yes	Hand Milking	Concrete	Weekly	Not clean
L	Lahore	14	1	13	2	1	10	Yes	Yes	Yes	Hand Milking	Concrete	weekly	Not clean

Supplementary Table S2: Animals Data: highlighted data shows samples processed for 16S r RNA gene base metagenomics

Farm Reference ID	Milk Sample ID	Metagenomic Ref #	Age	Parity	Lactation stage	Milk yield	Mastitis history	Antibiotic history	Date antibiotic given	Physical examination udder/milk	CMT Score	Somatic cell Count (SCC) X(1000)	Surf field test	Animal Status
I	M-75		4	1	early	8	Nil	Nil	Nil	Normal	T	227	Negative	Sub Clinical
I	M-76		4	1	early	7	Nil	Nil	Nil	Abnormal	2	567	Positive	Clinical
I	M-77	BM-77	6	2	early	5	Yes	Nil	Nil	Abnormal	2	454	Positive	Clinical
I	M-78		5	1	early	8	Yes	Nil	Nil	Normal	N	145	Negative	Healthy
I	M-79	BM-79	6	2	mid	5	Nil	Nil	Nil	Abnormal	1	413	Positive	Clinical
I	M-80	BS-80	4	1	mid	7	Nil	Nil	Nil	Normal	1	236	Positive	Sub Clinical
I	M-81	BM-81	4	1	early	6	Nil	Nil	Nil	Abnormal	1	388	Positive	Clinical
I	M-82	BM-82	5	2	mid	5	Yes	Nil	Nil	Abnormal	2	511	Positive	Clinical
I	M-83	BH-83	6	2	mid	8	Nil	Nil	Nil	Normal	N	78	Negative	Healthy
I	M-84	BS-84	5	2	early	8	Nil	Nil	Nil	Normal	T	213	Positive	Sub Clinical
J	M-85	BH-85	4	1	early	8	Yes	Nil	Nil	Normal	N	96	Negative	Healthy
J	M-86	BH-86	6	2	early	9	Nil	Nil	Nil	Normal	N	111	Negative	Healthy
J	M-87	BH-87	6	2	early	8	Nil	Nil	Nil	Normal	N	103	Negative	Healthy
J	M-88	BS-88	5	1	mid	8	Yes	Nil	Nil	Normal	1	278	Positive	Sub Clinical
J	M-89	BH-89	5	1	mid	9	Nil	Nil	Nil	Normal	N	123	Negative	Healthy
J	M-90	BS-90	6	2	early	8	Yes	Nil	Nil	Normal	1	249	Positive	Sub Clinical
J	M-91	BS-91	5	1	mid	4	Nil	Nil	Nil	Normal	1	265	Positive	Sub Clinical
J	M-92		4	1	late	7	Nil	Nil	Nil	Normal	N	98	Negative	Healthy
J	M-93		5	2	late	8	Nil	Nil	Nil	Normal	N	151	Negative	Healthy
J	M-94		6	2	late	5	Nil	Nil	Nil	Abnormal	2	567	Positive	Clinical
J	M-95	BM-95	6	2	early	4	Nil	Nil	Nil	Abnormal	2	424	Positive	Clinical
K	M-96		6	2	early	7	Nil	Nil	Nil	Normal	1	277	Positive	Sub Clinical
K	M-97		4	1	early	7	Nil	Nil	Nil	Normal	1	287	Positive	Sub Clinical
K	M-98		4	1	late	8	Nil	Nil	Nil	Normal	N	151	Negative	Healthy
K	M-99		4	1	early	9	Nil	Nil	Nil	Normal	N	165	Negative	Healthy
K	M-100		5	2	late	7	Nil	Nil	Nil	Normal	1	300	Positive	Sub Clinical
K	M-101		6	2	early	6	Yes	Nil	Nil	Abnormal	2	590	Positive	Clinical
K	M-102		6	2	late	5	Yes	Nil	Nil	Abnormal	2	600	Positive	Clinical

L	M-103		5	2	mid	5	Nil	Nil	Nil	Abnormal	2	470	Positive	Clinical
L	M-104		4	1	early	7	Nil	Nil	Nil	Normal	T	241	Positive	Sub Clinical
L	M-105		5	2	late	6	Nil	Nil	Nil	Normal	1	297	Positive	Sub Clinical
L	M-106		6	1	late	7	Nil	Nil	Nil	Normal	T	229	Positive	Sub Clinical
L	M-107		5	2	early	9	Yes	Nil	Nil	Normal	N	110	Negative	Healthy
L	M-108		4	1	mid	8	Nil	Nil	Nil	Normal	N	67	Negative	Healthy
L	M-109		6	2	early	6	Nil	Nil	Nil	Abnormal	2	432	Positive	Clinical
L	M-110		4	1	mid	5	Nil	Nil	Nil	Abnormal	1	412	Positive	Clinical

CMT(California Mastitis Test) score= N= Negative, T=trace, 1= weak positive, 2= and above = Distinct Positive

Clinical=Clinical Mastitis, Subclinical=Subclinical Mastitis

The Colored rows indicates the samples processed for 16S rRNA gene base metagenomic analysis

=Healthy, =

=Clinical Mastitis

=Subclinical Mastitis

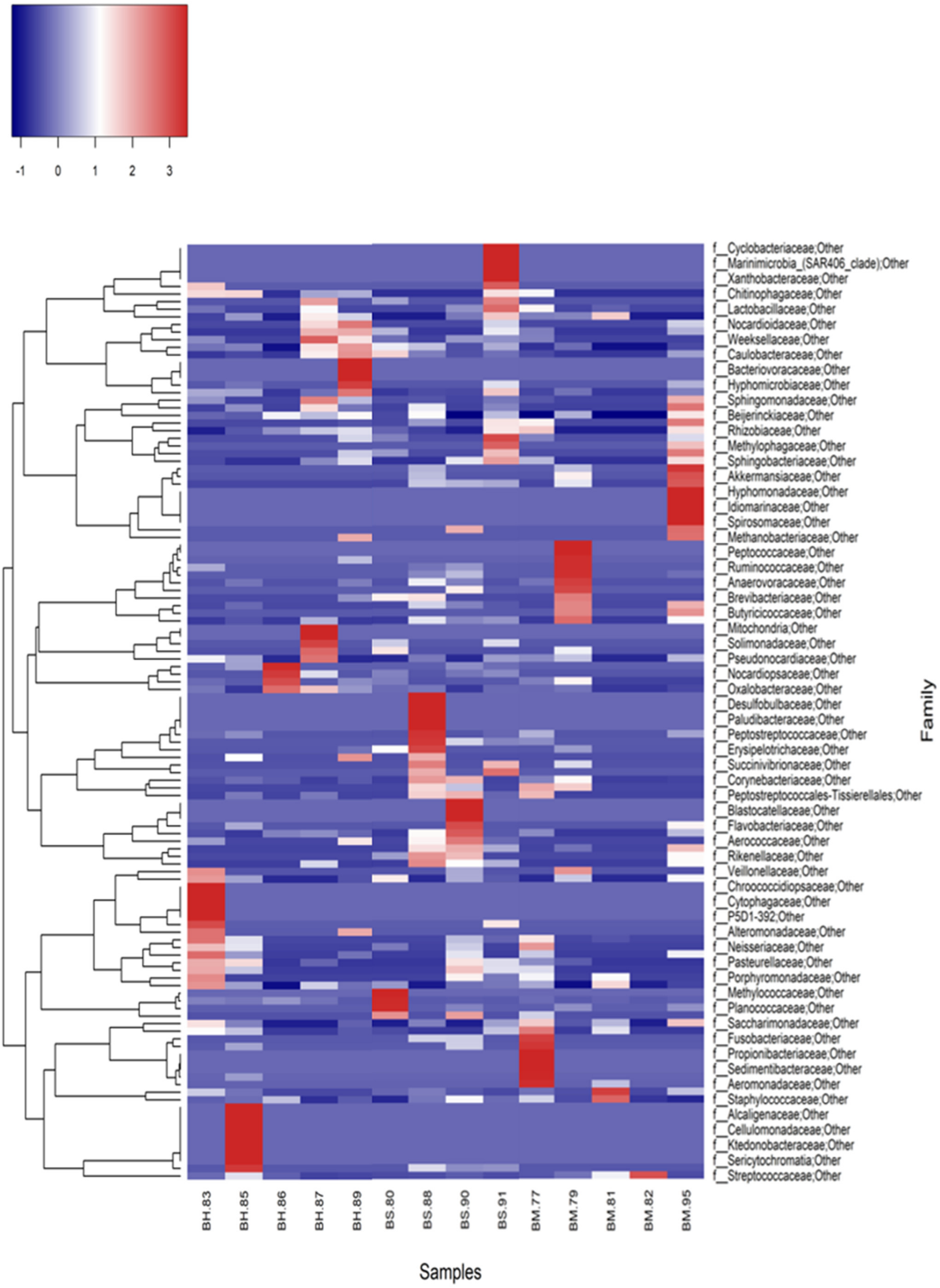

Figure S1: Represnation of family level taxonomical composition in individual samples through heat map (Intensity of color shows the realtive abundance of phyla in milk microbiota of Nili Ravi buffalo with different udder health status)

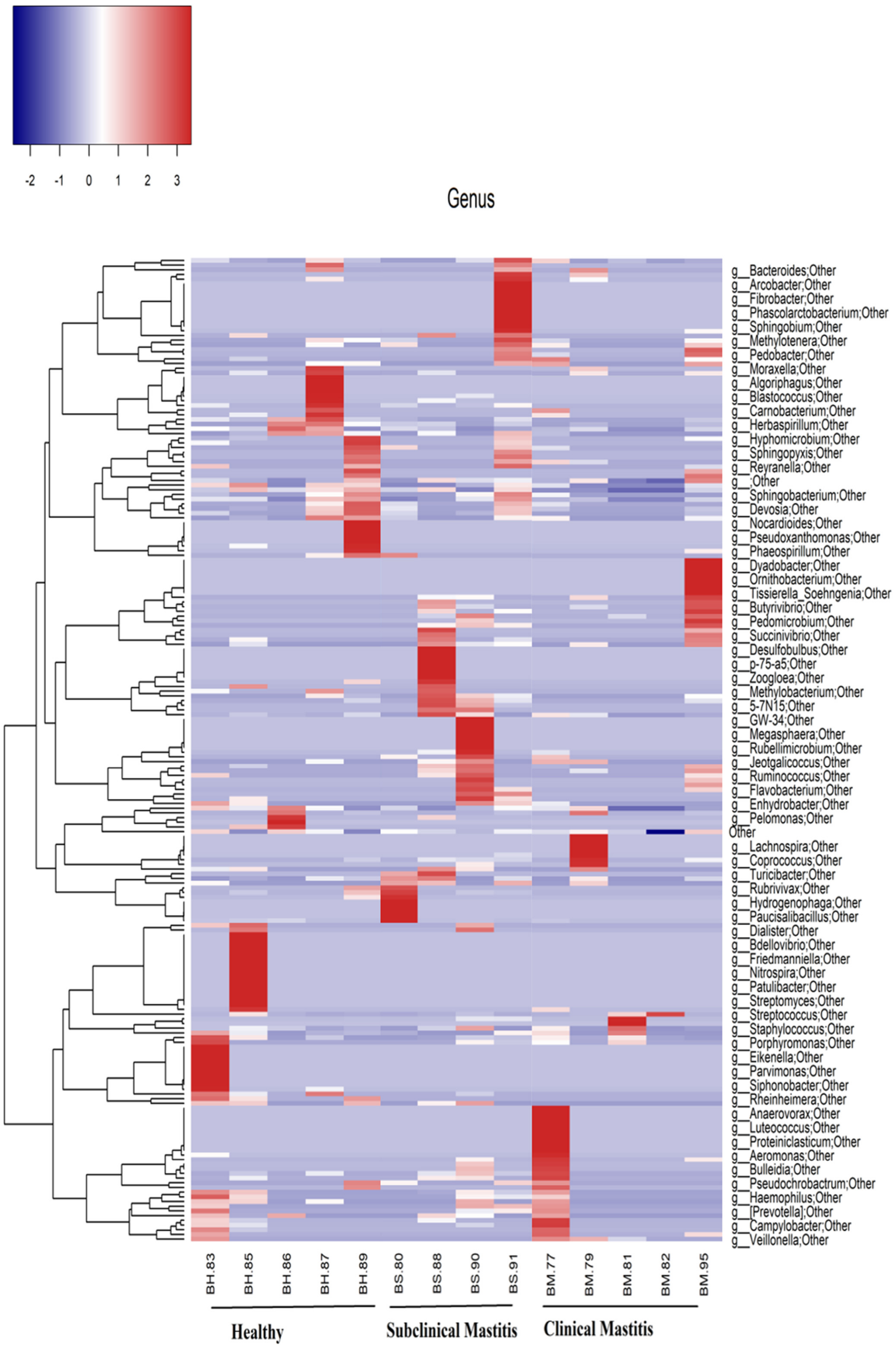

Figure S2: Represnation of genus level taxonomical composition in individual samples through heat map (Intensity of color shows the realtive abundance of phyla in milk microbiota of Nili Ravi buffalo with different udder health status)

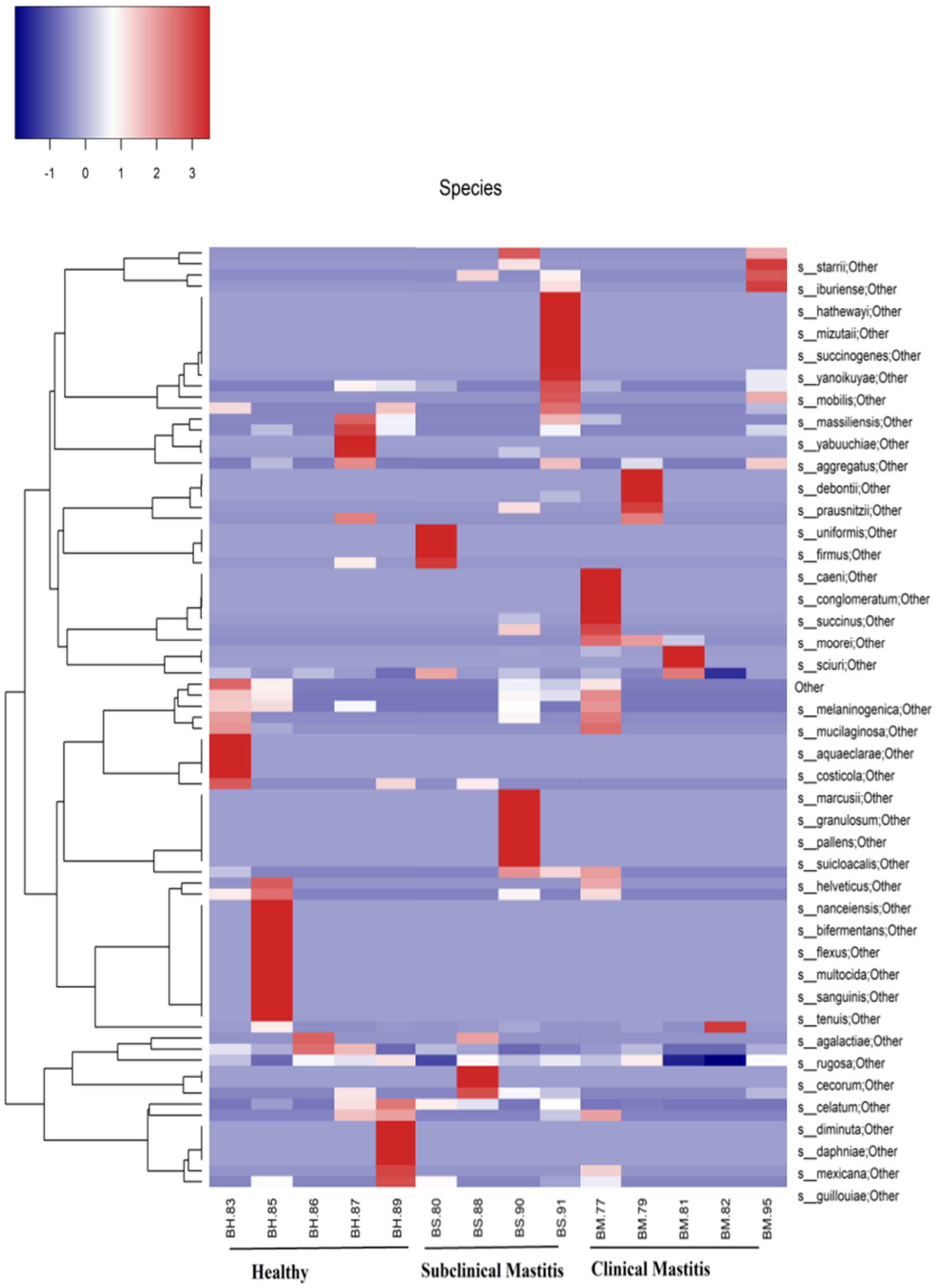

Figure S3: Represnation of Species level taxonomical composition in individual samples through heat map (Intensity of color shows the realtive abundance of phyla in milk microbiota of Nili Ravi buffalo with different udder health status)

Supplementary Table S3: Concentration of extracted DNA from milk samples

Metagenomic ID.	Concentration(µg/ml)
BM-77	29.7
BM-79	14.3
BS-80	13.2
BM-81	28.3
BM-82	70.9
BH-83	43.2
BS-84	39.2
BH-85	28.3
BH-86	26.7
BH-87	36.0
BS-88	17.20
BH-89	40.6
BS-90	23.4
BS-91	46.5
BM-95	32.4
